# Experimental Investigation on the Wear and Damage Behaviors of Machined Wheel-Rail Materials under Dry Sliding Conditions

**DOI:** 10.3390/ma14030540

**Published:** 2021-01-23

**Authors:** Peijie Liu, Yanming Quan, Junjie Wan, Lang Yu

**Affiliations:** School of Mechanical and Automotive Engineering, South China University of Technology, Guangzhou 510640, China; meymquan@scut.edu.cn (Y.Q.); wjj2525625@163.com (J.W.); yulangyuxida@163.com (L.Y.)

**Keywords:** turning, grinding, wheel/rail contact, sliding, wear

## Abstract

Rail grinding and wheel turning can effectively remove surface defects and unevenness, which is a crucial process for the safe and smooth operation of trains. Machined surface integrity of wheel/rail materials significantly influences their tribological property. In this study, firstly, the rail blocks were ground via a cylindrical grinding machine, and the wheel rings were turned by a computer numerical control (CNC) lathe with varied parameters. Then, the sliding wear and damage characteristics of the machined wheel/rail samples under dry conditions were studied by virtue of a block-on-ring tribometer. The results show that the surface microhardness of the ground rail blocks is larger than that of wheel rings, while the surface roughness and the thickness of the subsurface plastic deformation layer (SPDL) of rail blocks are much smaller than those of wheel rings. After sliding, the surface microhardness of wheel/rail samples increases remarkably. The thickness of the SPDL, the wear loss, and the increase degree of surface microhardness of rail blocks are larger than those of wheel rings. Surface microhardness, roughness and the SPDL of the machined wheel/rail samples impose a combined influence on the anti-wear property, and the tribological pair with proper initial surface roughness and microhardness engenders the smallest amount of total wear loss.

## 1. Introduction

Wheel and rail are extremely crucial infrastructures in the railway network, which exerts a decisive influence on the reliable and secure operation of the railway transportation system. The ever-increasing train speed and traffic volume bring a series of challenges to the railway industry due to the fact that the flaws generated by the dynamic and intricate wheel/rail interaction are more likely to happen under harsher and more complex service conditions, such as surface spalling [1], fatigue cracks [2], head checks [3] and squats [4], etc. The aforementioned defects seriously threaten the security of the railway system if they are not eliminated in time, and this can finally bring about the partial or complete failure of wheel/rail system. In the railway maintenance industry, owing to the structural difference between the wheel and the rail, different machining methods need to be utilized to reprofile the wheel and the rail. Aiming at eradicating the unfavorable diseases existing on the surfaces of wheel/rail materials, it has been increasingly vital to carry out regular wheel/rail maintenance practices (namely, rail grinding and wheel turning) to restore their profiles, which can guarantee the proper and secure wheel/rail contact. Rail grinding technology has been adopted by the railway industry to reprofile the rail head by virtue of grinding stones, during which the surface flaws and irregularity can be eradicated effectively via peripheral grinding method [5,6]. Similarly, wheel turning practice is employed to eliminate the diseases existing on the surface of wheel tread and flange so as to prolong the service life span [7]. Wheel turning and rail grinding techniques have huge effects on the steady and reliable running of trains, which is conducive to ameliorate the wheel/rail interaction conditions.

Currently, the studies on the friction and wear performance of wheel/rail materials primarily concentrate on the rolling-sliding contact mode, and the research contents mainly center on the hardness matching of wheel/rail materials [8,9,10], diverse wheel/rail creepages [11,12], selection of wheel/rail materials [13,14], laser treatment of wheel/rail materials [15,16,17] and different lubrication conditions [18,19]. Razhkovskiy et al. [9] experimentally obtained the wheel/rail wear and damage data, and they found that the optimal range of the wheel-rail material hardness ratio H_w_/H_r_ is (0.91–0.97):1, or close to 1:1. Wang et al. [12] investigated the impact of creepage in the propagation of rolling contact fatigue (RCF)-related cracks of rail material under wet conditions, and they concluded that the worn surface morphology of the studied material shifts from slight surface fatigue crack to serious fatigue crack and pitting as the creepage changes from 0 to 10%. Stock et al. [14] studied the wear and RCF characteristics of bainitic and pearlitic rail steels, and they found that the wear resistance of bainitic steels is weakened compared with the pearlitic steel with the same hardness level. Roy et al. [16] studied the effect of deposition material and heat treatment on the wear behaviors of laser-cladded rail materials, and they found that SS420 (i.e., a kind of high strength martensitic steel with excellent abrasion-resistance performance) cladding possessed the best wear-resistance property. Compared with the studies on the wheel/rail rolling-sliding wear behaviors, much fewer literatures focusing on the wear and damage features under pure sliding condition are available. Robles Hernández et al. [20] carried out an in-depth comparison on the results of the ball-on-disk sliding tests using the small rail samples and a full-scale actual rail workpiece, and the experimental results revealed that wear performance has a good correlation with the material flow and work hardening behavior. Khalladi et al. [21] studied the tribological characteristics of wheel-rail materials under various contaminants via a pin-on-disk tribometer, and they found that, compared with the clean and lubricated conditions, all the contaminants increased the friction coefficient, and the sand contaminant generates a larger friction coefficient. Furthermore, there are also very few systematic researches addressing the sliding wear and damage behaviors of machined wheel/rail materials, which indicates that there is a lack of adequate research on the influence of surface integrity of the wheel/rail materials after machining on the wear behaviors. As a matter of fact, the surface integrity of the workpiece after machining directly influences the tribological behavior during sliding. In our previous work, we conducted a systematic investigation on the rolling-sliding wear behaviors of machined wheel/rail materials, and we found that the rail material displays better anti-wear properties and that a thicker SPDL of the rail samples was formed [22,23].

In this study, firstly, the rail blocks are ground via a cylindrical grinding machine and the wheel rings are turned by a CNC lathe with varied machining parameters, which aims to acquire the corresponding surface integrity. The machined surface microhardness, roughness and the thickness of the SPDL are measured. Then, the sliding wear and damage characteristics of the machined wheel/rail samples under dry condition are systematically investigated by virtue of a block-on-ring tribometer by altering various tribological pairs. According to the experimental results, detailed analyses are conducted on the friction coefficient, wear loss and surface microhardness of the wheel/rail samples. Additionally, the microscopic characterization of the worn surface morphology and the SPDL of the wheel/rail samples after sliding is performed.

## 2. Materials and Methods

### 2.1. Materials

**Figure 1:**
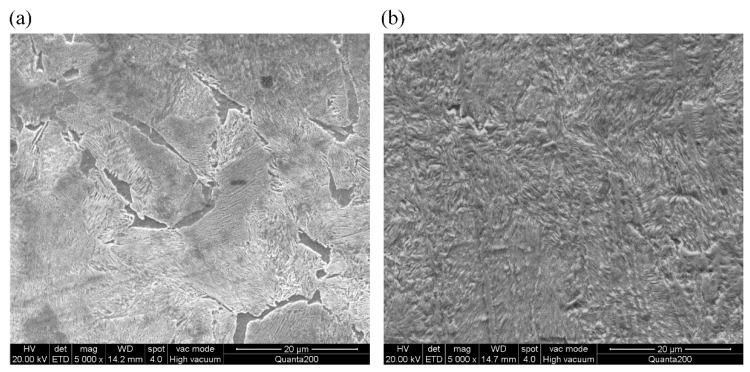
Microstructures of wheel/rail samples: (**a**) wheel steel; (**b**) rail steel.

The experimentally-employed rail material (Chinese brand: U71Mn, manufactured by Pangang Group Panzhihua Steel & Vanadium Co., Ltd., Panzhihua, Sichuan, China) is a kind of Mn-steel made from high carbon steel to possess high fatigue toughness, which is extensively utilized in current Chinese railway network [24]. The experimentally-utilized wheel material (Chinese brand: CL60, which was manufactured by Maanshan Iron & Steel Co., Ltd., Maanshan, Anhui, China) is one of the commonly-employed solid forged Mn-steels in current Chinese railway network [22]. Table 1 presents the chemical constituents of the studied materials. The representative microstructures of U71Mn and CL60 are presented in Figure 1 by virtue of a scanning electron microscope (SEM, Quanta 200, FEI, Eindhoven, The Netherlands), from which it can be seen that CL60 exhibits the ferrite-pearlite microstructure, and the microstructure U71Mn consists of pearlite [22].

### 2.2. Experimental Procedure

**Figure 2:**
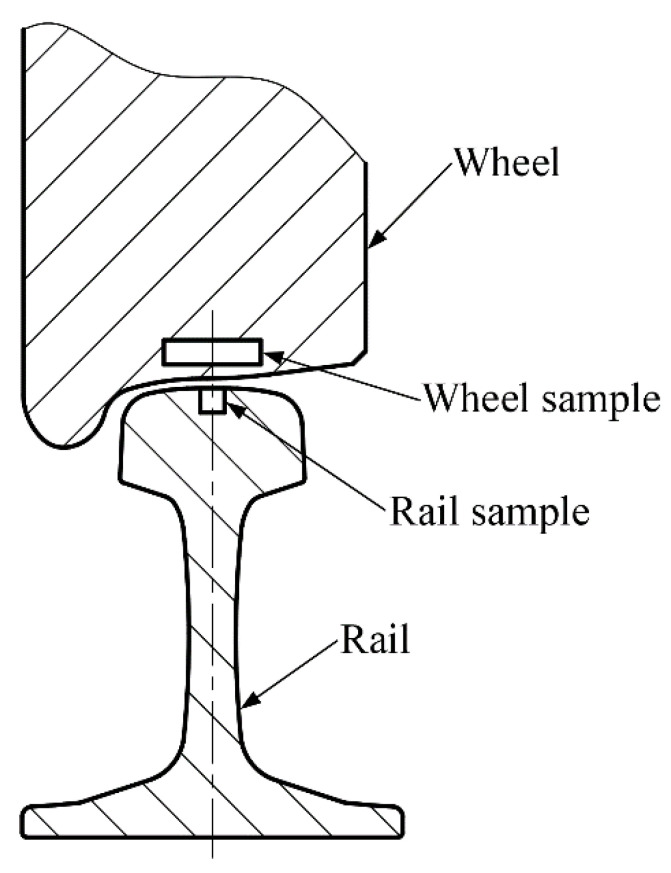
Sampling diagram of wheel/rail specimens.

**Figure 3:**
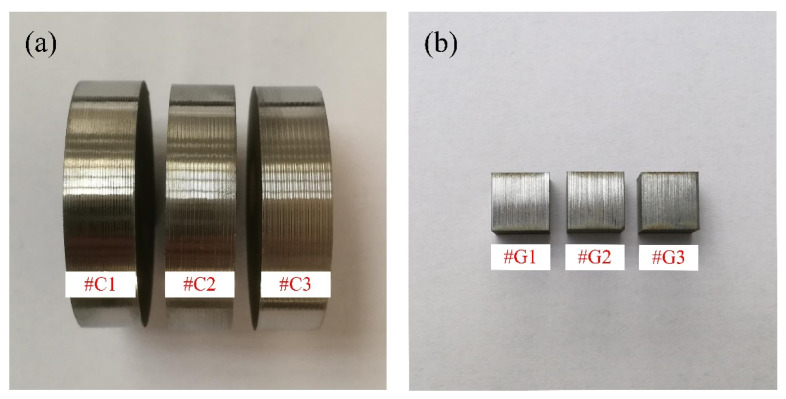
Photos of the machined wheel/rail specimens: (**a**) turned wheel specimens; (**b**) ground rail specimens.

Prior to the implementation of sliding friction and wear tests, the surface of the rail workpiece was machined by virtue of a reliable self-developed rail form cylindrical grinding experimental platform with varied grinding parameters, and an electroplated diamond grinding wheel with nickel-cobalt being the metal binder was employed. The abrasive grain size of the grinding wheel is 80 mesh. After form grinding the rail workpiece, the rail specimens were directly sampled right in the middle of ground rail head by wire electric discharge machining (WEDM), as shown in Figure 2. The machining method and machining parameters of the wheel samples utilized in this study prior to the sliding tests are the same as those presented in our previous work [22]. The wheel rollers were sampled by WEDM with their top surfaces adjacent to the surface of wheel tread, and then these sampled wheel rollers were turned via a CNC lathe (CKA6150i, DMTG Co. Ltd., Dalian, Liaoning, China) with different machining parameters. During the turning tests, the indexable coated carbide cutting inserts (GC4215, Sandvik AB, Stockholm, Sweden) were engaged. Table 2 and Table 3 list the specific machining parameters of the studied wheel/rail samples, respectively. During the machining process, the adopted parameters are consistent with the parameter ranges performed by the real wheel/rail reprofiling field. The photographs of the machined specimens are shown in Figure 3. A surface profilometer (RTEC-UP Dual Mode, San Jose, CA, USA) was utilized to inspect the surface roughness *R*_a_. A Vickers hardness tester (HV-1000, Shanghai, China) was employed to measure the surface microhardness HV_0.1_ of the machined samples. Table 4 lists the averaged measurement results, from which it can be seen that the values of surface microhardness HV_0.1_ of the wheel/rail specimens after machining all display an upward trend. Due to the same processing method and parameters of the turned wheel samples, the values of surface roughness and hardness of the turned wheel samples in Table 4 in this study are identical to those reported in our previous work [22]. The machining method and machining parameters of the rail samples utilized in this study prior to the sliding tests are different from those presented in our previous work; therefore, the values of surface roughness and hardness of the ground rail samples in Table 4 in this study are different from those reported in our previous work [22]. The values of surface roughness *R*_a_ of the turned wheel rings exhibit a conspicuous increasing trend, by contrast, the increase degree of *R*_a_ of the ground rail blocks is significantly smaller.

**Figure 4:**
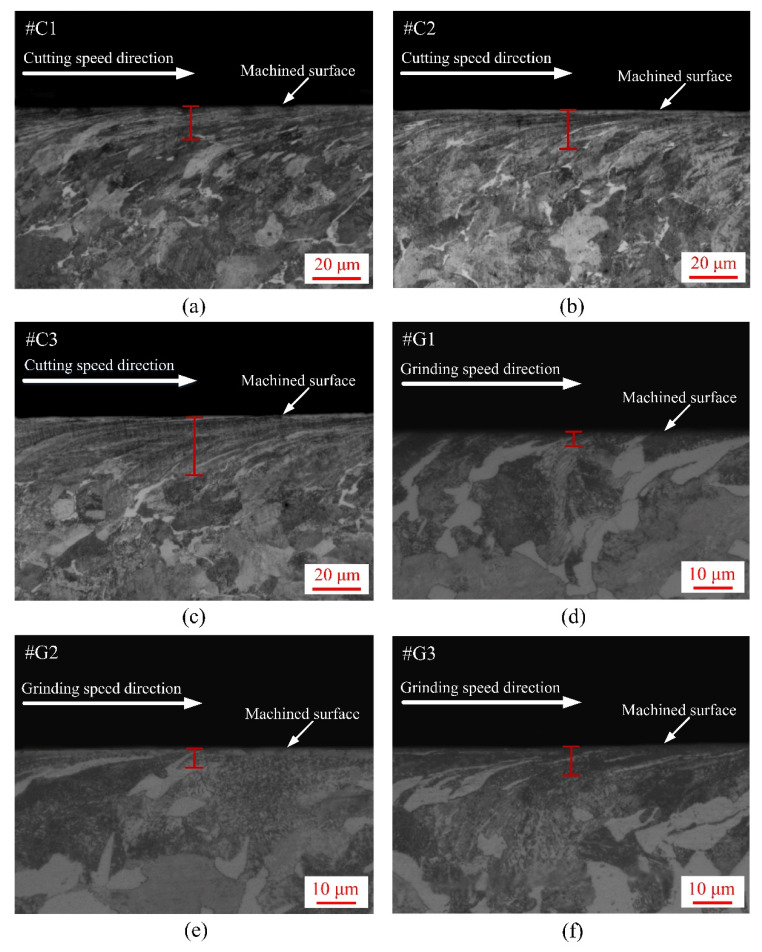
Micrographs of SPD of the machined wheel rings and rail blocks: (**a**) #C1; (**b**) #C2; (**c**) #C3; (**d**) #G1; (**e**) #G2; (**f**) #G3.

The subsurface plastic deformation (SPD) of the turned wheel rings and ground rail blocks were characterized by virtue of an optical microscope (i.e., OM, DMI5000 M, Leica, Wetzlar, Germany) after the procedures of abrasive paper grinding, fine polishing and etching, as presented in Figure 4, from which it can be found that thickness of the SPDL of the ground rail blocks is much smaller than that of the turned wheel rings prior to the sliding experiment. Conspicuous SPD of the turned wheel rings #C1, #C2 and #C3 is observed, and their thickness of the SPDL falls in between 14 and 25 µm, which displays an upward trend. The thickness of the SPDL of the ground rail blocks #G1, #G2 and #G3 shows a slight upward trend, which falls in between 3 and 7 µm. By contrast, the thickness increase degree of the SPDL of the turned wheel rings is much greater than that of the ground rail blocks.

**Figure 5:**
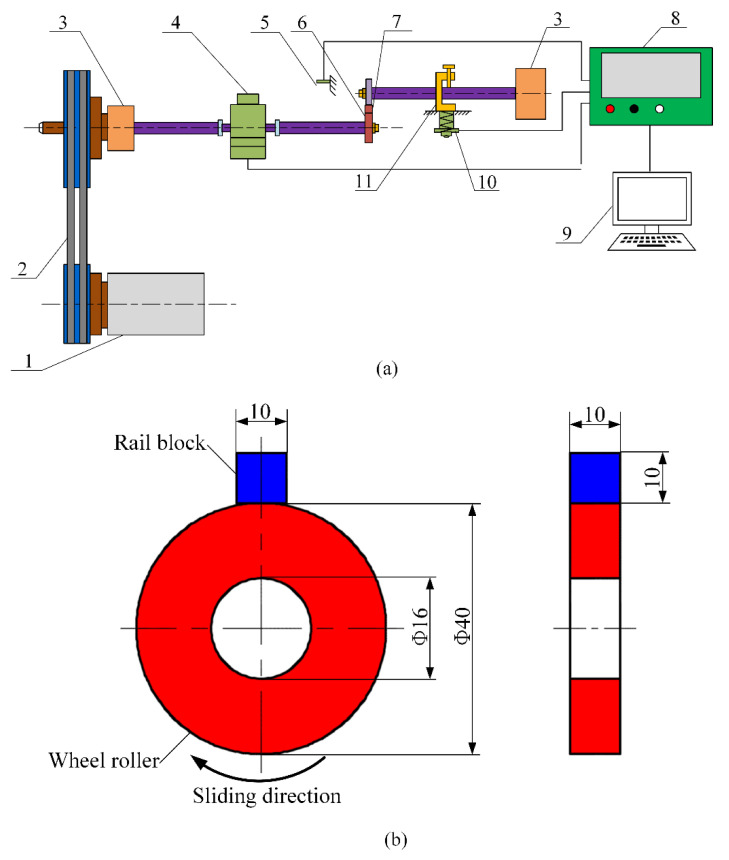
Tribometer used for the sliding wear experiments. (**a**) Schematic of the tribometer. 1, DC motor; 2, driving belt; 3, gearbox; 4, torque sensor; 5, rotational velocity sensor; 6, lower ring; 7, upper block; 8, control cabinet; 9, computer; 10, load sensor; 11, compression spring. (**b**) Configuration and dimension of the wheel ring and rail block (unit: mm).

The sliding friction and wear experiments were carried out under dry conditions via a block-on-ring tribometer, which is presented in Figure 5a. The tribometer (MM2000, Jinan, Shandong, China) used for the sliding tests is comprised of an upper block (i.e., rail block) and a lower ring (i.e., wheel ring or roller). The rail block remains stationary during the sliding process, and the wheel ring is activated and controlled by a DC motor with the propelling of the driving belt and gearbox, which finally makes a sliding contact. The loading force executed on the rail block is determined by adjusting the compression spring. A torque sensor is utilized to measure the frictional torque. The loading value, friction coefficient, rotational speed and duration were recorded and measured by a computerized system. When installing the samples, the surface groove direction of the ground rail block is parallel to the rolling direction of the wheel ring. The detailed configuration and dimension of the wheel ring and rail block are illustrated in Figure 5b. The outer diameter of all the wheel rings is 40 mm, and all the rail blocks have the size of 10 mm × 10 mm × 10 mm. The line contact width is 10 mm. In this study, the rotational velocity of the wheel ring was set as 400 r/min, and the number of rolling cycles was 3.2 × 10^4^. The loading value was set as 200 N.

To perform the sliding friction and wear tests under dry condition, the turned wheel rings #C1, #C2 and #C3 and the ground rail blocks #G1, #G2 and #G3 mentioned above are paired with each other, which constitutes nine tribological pairs altogether, namely, #C1-#G1, #C2-#G1, #C3-#G1, #C1-#G2, #C2-#G2, #C3-#G2, #C1-#G3, #C2-#G3 and #C3-#G3.

**Figure 6:**
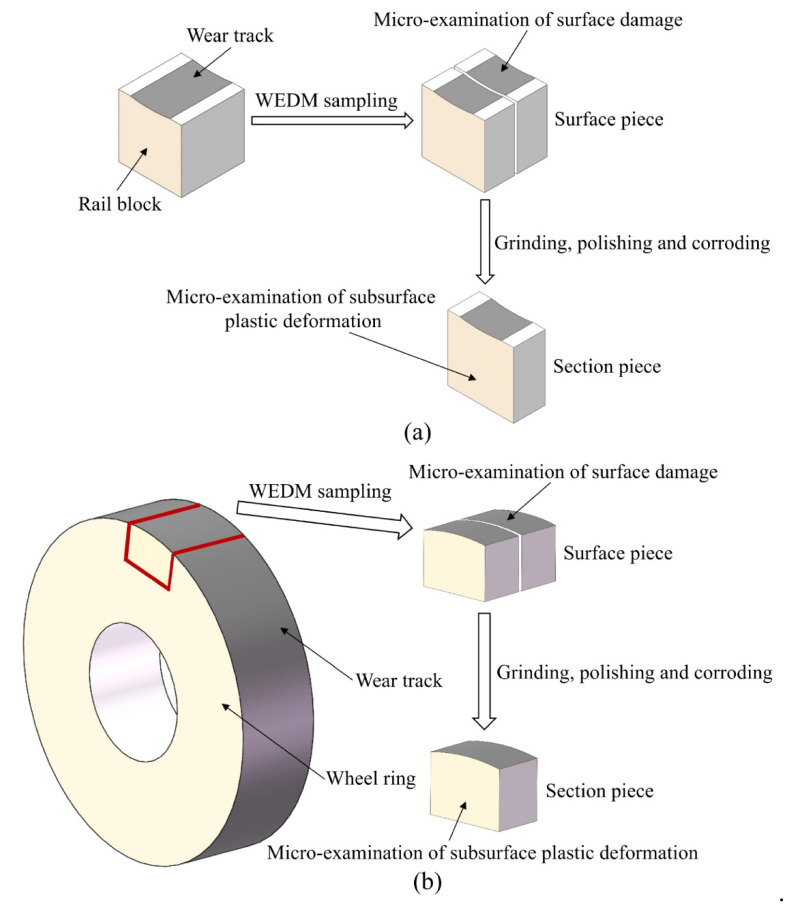
Sampling preparation for the characterization of circumferential surface and section of the wheel/rail samples after sliding test: (**a**) sampling preparation of the rail samples; (**b**) sampling preparation of the wheel samples.

Before and after each sliding test, all the wheel rings and rail blocks were ultrasonically and meticulously cleaned in an ethanol bath for 10 min and then were dried by an air blower. The surface microhardness of each wheel/rail sample was measured, and every sample was weighed by an analytical balance with the measurement precision of 0.1 mg. The sampling preparation for the characterization and measurement of wheel/rail specimens after the sliding test is shown in Figure 6. A scanning electron microscope (SEM, Quanta 200, FEI, Eindhoven, The Netherlands) was used to characterize the worn surfaces. After being mounted, ground with wet abrasive papers, carefully polished and etched with 4% natal, the cross section of the wheel/rail specimens was micro-observed.

## 3. Results and Discussion

### 3.1. Friction Coefficient

**Figure 7:**
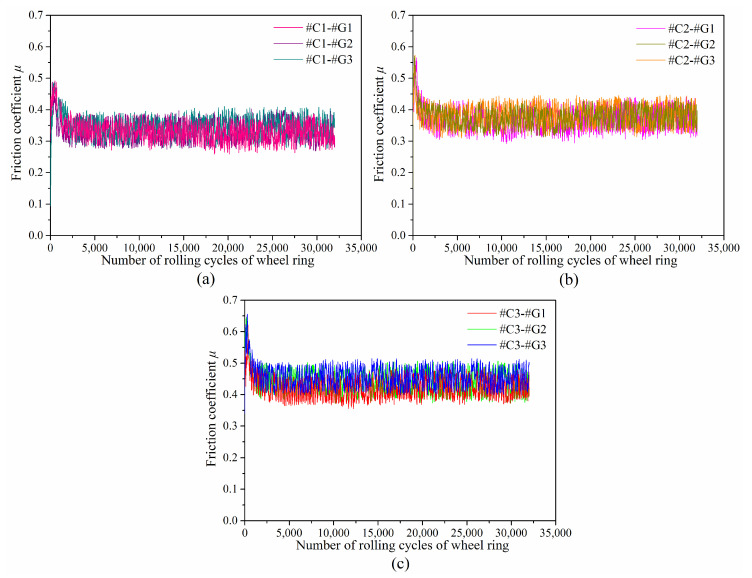
Variations of friction coefficients between various tribological pairs, as a function of wheel ring rolling cycles: (**a**) #C1-#G1, #C1-#G2 and #C1-#G3; (**b**) #C2-#G1, #C2-#G2 and #C2-#G3; (**c**) #C3-#G1, #C3-#G2 and #C3-#G3.

Figure 7 displays the variations of friction coefficients as a function of rolling cycles of wheel ring. It can be found that the friction coefficient between different wheel/rail tribological pairs fluctuates greatly, and the changing law is similar. The variations of friction coefficients are classified into two phases, which includes the break-in phase and the steady-wear phase. At the very early stage of the sliding test, there is a sharp increase in the friction coefficient due to the difference of the initial surface state between the tribological pair, which indicates that the original surface roughness of wheel/rail samples engenders a momentary friction rise. When the friction coefficient reaches its peak value, it gradually decreases and eventually steps into the stable wear stage. This is attributed to the reason that the micro-peaks on the surface are worn off little by little as the sliding test proceeds, then the stable wear phase forms when the formation rate of the wear debris detached from the tribological pair and the material falling rate off the surface of wheel/rail samples reach a dynamic balance state. It takes approximately 1700 r of the rolling cycle of the wheel ring to complete the translation from the break-in phase to stable wear phase. At the stable wear stage, the friction coefficients among tribological pairs #C1-#G1, #C1-#G2 and #C1-#G3 range from 0.32 ± 0.06 to 0.34 ± 0.06, the friction coefficients among tribological pairs #C2-#G1, #C2-#G2 and #C2-#G3 range from 0.37 ± 0.07 to 0.39 ± 0.07, and the friction coefficients among tribological pairs #C3-#G1, #C3-#G2 and #C3-#G3 range from 0.42 ± 0.07 to 0.45 ± 0.06.

The previous investigations revealed that surface microhardness of wheel/rail materials makes little difference on the friction coefficient of the tribological pairs [25,26], which reveals that the friction coefficient is primarily dominated by the original surface roughness of the wheel/rail samples. Compared with Figure 7a,c, when pairing with the rail blocks ground with the same parameters, the corresponding friction coefficient of #C3 is the largest, and the corresponding friction coefficient of #C1 is the smallest, which is caused by the larger original surface roughness of #C3 than that of #C1 and #C2.

### 3.2. Surface Hardness and Wear Loss

**Figure 8:**
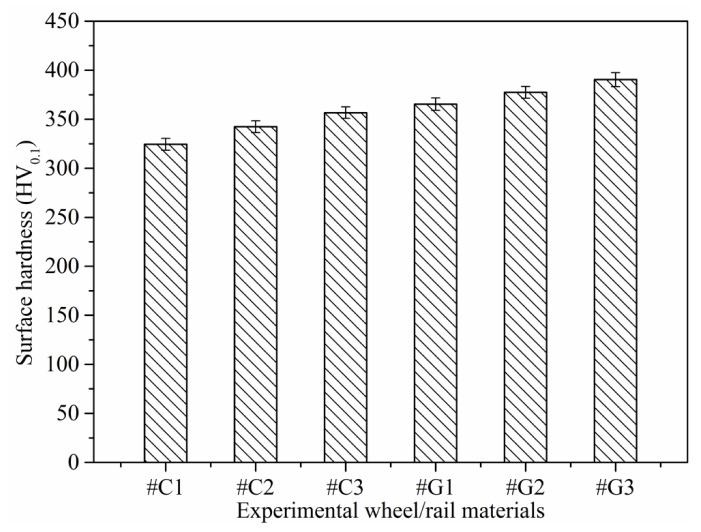
Surface microhardness of the turned wheel rings and ground rail blocks before sliding.

**Figure 9:**
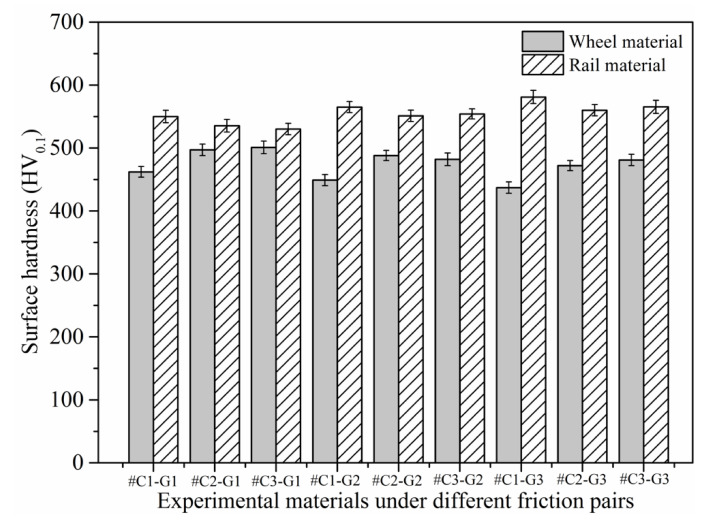
Variations of surface microhardness of the wheel rings and rail blocks after sliding.

Figure 8 depicts the surface microhardness of wheel/rail samples prior to the sliding test. The variations of surface microhardness of wheel/rail samples after sliding is presented in Figure 9. It can be seen from Figure 8 and Figure 9 that the surface microhardness of the wheel rings and rail blocks increases remarkably after sliding. In addition, the surface microhardness of the rail blocks is larger than that of the wheel rings before and after the sliding test, which is similar to the phenomenon under the dry rolling-sliding condition reported in [22]. The surface microhardness of the wheel rings prior to sliding falls in between 324.48 and 356.72 HV_0.1_, and it ranges from 437.34 to 501.09 HV_0.1_ after sliding. The maximal surface microhardness increment of the wheel rings is 45.2%. Meanwhile, before the sliding test, the surface microhardness of the rail blocks ranged from 365.12 to 390.41 HV_0.1_, and it ranges from 530.16 to 581.84 HV_0.1_ after sliding. The maximal surface microhardness increment of the rail blocks is 50.8%. Generally speaking, the surface microhardness increase degree of the wheel rings is smaller that of the rail blocks after sliding. It can be found that, whether it is under the dry sliding condition or under the dry rolling-sliding condition, the increase degree of surface microhardness of the rail samples is larger than that of the wheel samples after each friction and wear test. When pairing with the rail blocks ground with the same parameters, the values of the surface microhardness of #C2 and #C3 are similar, and they are greater than that of #C1, which indicates that the wheel rings with smaller initial surface roughness and surface microhardness after turning engenders the comparatively small surface microhardness after the sliding contact. When pairing with the wheel rings turned with the same parameters, the surface microhardness value of #G3 is larger than that of #G1 and #G2, which reveals that the surface microhardness of the rail blocks with larger original surface microhardness after grinding enhances accordingly after sliding, and this is conducive to decrease the wear loss of rail material.

**Figure 10:**
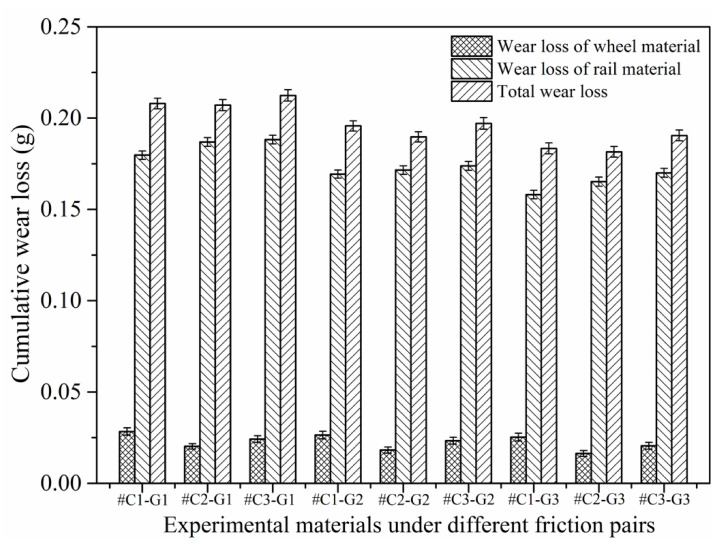
Cumulative wear loss of the wheel rings and rail blocks after sliding.

Figure 10 displays the cumulative wear loss of the wheel rings and rail blocks after the sliding contact, from which it can be found that the wear loss of the rail material after every sliding test is above 0.1581 g, while the wear loss of the wheel material after each sliding test falls in between 0.0163 and 0.0283 g, which means that the wear loss of the rail material is much larger than that of the wheel material. The wheel/rail wear loss under the dry sliding condition is totally different from that presented in our previous work [22], the wear loss of the wheel material is much larger than of the rail material under the dry rolling-sliding condition (namely, the rail material exhibits better anti-wear performance), which is just the opposite of the results of wheel/rail wear loss obtained under the dry sliding condition. The difference in the wheel/rail wear loss results is caused by the various utilized frictional modes. During the sliding process, the rail block makes constant contact with the surface of the wheel ring, and the rail materials are worn off continuously, while the circumferential surface of the wheel ring makes cyclic and intermittent contact with the rail block, resulting in smaller wear loss of the wheel material. Under the sliding condition, the wheel material exhibits a better wear-resistance property. When #C1, #C2 and #C3 pair with the rail blocks ground with the same parameters, the wear loss of #C1 is larger than that of #C2 and #C3, while the wear loss of #C2 is slightly smaller that of #C3, which reveals that the surface microhardness and surface roughness of the machined wheel/rail material impose a comprehensive influence on their abrasion-resistance performance. The surface microhardness of #C3 is higher than that of #C2, but the surface roughness of #C3 is larger than that of #C2, which can make the wear of #C3 faster, finally giving rise to larger wear loss than that of #C2 under the combined effect of surface microhardness and roughness. When #G1, #G2 and #G3 pair with the wheel rings turned with the same parameters, the wear loss of #G1 is the largest, followed by #G2, and then by #G3. The reason for this is that there is no conspicuous discrepancy of surface roughness amongst the ground rail blocks; thus, the dominating factor influencing the wear loss of rail blocks is the original surface microhardness. In view of the total wheel/rail wear loss among all the tribological pairs, #C2-#G1, #C2-#G2 and #C2-#G3 exhibit the comparatively small total amount of wheel/rail wear loss after the sliding contact, which reveals that the comprehensively preferable wear-resistance performance is achieved as #C2 pairs with the rail blocks.

### 3.3. Surface Damage Characteristics

**Figure 11:**
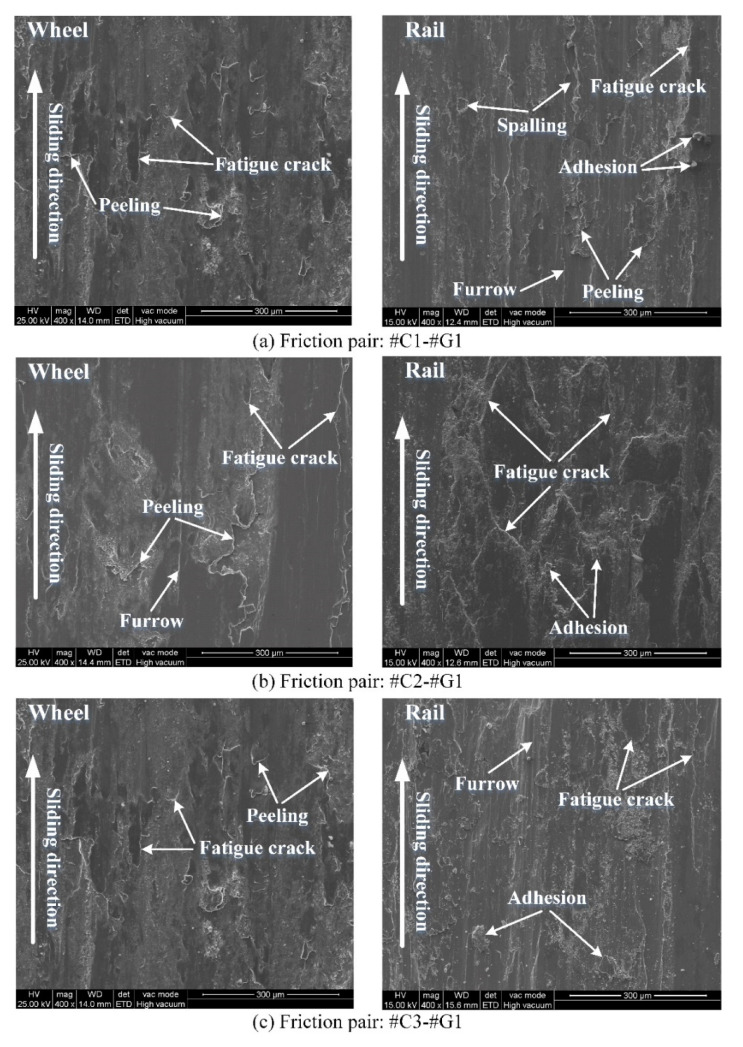
Morphology of worn surface of the wheel/rail samples under varied tribological pairs after the sliding contact: (**a**) #C1-#G1; (**b**) #C2-#G1; (**c**) #C3-#G1.

The worn surface morphology of the wheel/rail samples under tribological pairs #C1-#G1, #C2-#G1 and #C3-#G1 is presented in Figure 11, from which it can be observed that there are discrepancies on the worn surface damage morphologies among the rail blocks and wheel rings. By and large, more serious worn surface damage of the rail blocks can be observed, which is different from the experimental results under dry rolling-sliding conditions reported in our previous work [22]. Under the dry rolling-sliding condition, the surface damage of the wheel samples is more serious than that of the rail samples. The worn surface morphology of the wheel samples is dominated by the combination of adhesive wear, spalling, peeling and fatigue cracks, while the primary surface damage of the rail specimens displays obvious pitting and fatigue cracks. In this study, the worn surface morphology of the wheel rings mainly presents peeling, adhesive wear and fatigue cracks under dry sliding conditions, while the worn surface morphology exhibits the combination of spalling, furrow wear, peeling, fatigue cracks and various degrees of adhesion. Spalling is characterized by the damage (i.e., falling-off material) caused by Hertzian contact fatigue, which leads to the formation of craters in the contact region. Furrow wear is characterized by the ploughing or scratching marks (namely, grooves) left on the surface of wheel/rail contact region, which usually results from the ploughing effect caused by the wear debris trapped within the wheel/rail contact interface. The direction of the ploughing grooves is usually parallel to the sliding direction. Adhesion refers to the smeared material which shifts from the surface of one material with lower hardness to the surface of another material with higher hardness between the tribological pair. The degree of adhesion hinges on the size and number of the smeared material within the wheel/rail contact region. Peeling is characterized by the material flaking that forms on the worn surface, which usually possesses the tongue-like shape. The end part of the peeling region is usually detached from the matrix of material, which forms an opening or gap between the peeled and matrix material. Fatigue cracks are characterized by the partial material fracture under continuous loading, and most crack ends are pointed, which can cause stress concentration. The damage degree of each type of the aforementioned worn surface morphology depends on the experimental conditions. Owing to larger surface roughness and smaller surface microhardness of the wheel rings, the adhesion of wheel materials is prone to appear within the wheel/rail contact region during the sliding contact; meanwhile, the adhesive points are sheared, shifted and smeared on the surface of rail blocks whose microhardness is larger, which eventually results in the adhesive wear of the wheel rings. After pairing with the rail blocks ground with the same parameters, compared with the worn surfaces of #C1, #C2 and #C3, densely and widely-distributed fatigue cracks and peeling can be found on the worn surfaces of #C1 and #C3, while relatively smoother surface of #C2 is visually apparent after the sliding contact. This reveals that when pairing with the rail blocks ground with same parameters, the turned wheel ring with smaller initial surface roughness does not guarantee to bring forth the corresponding worn surface with less damage after sliding.

**Figure 12:**
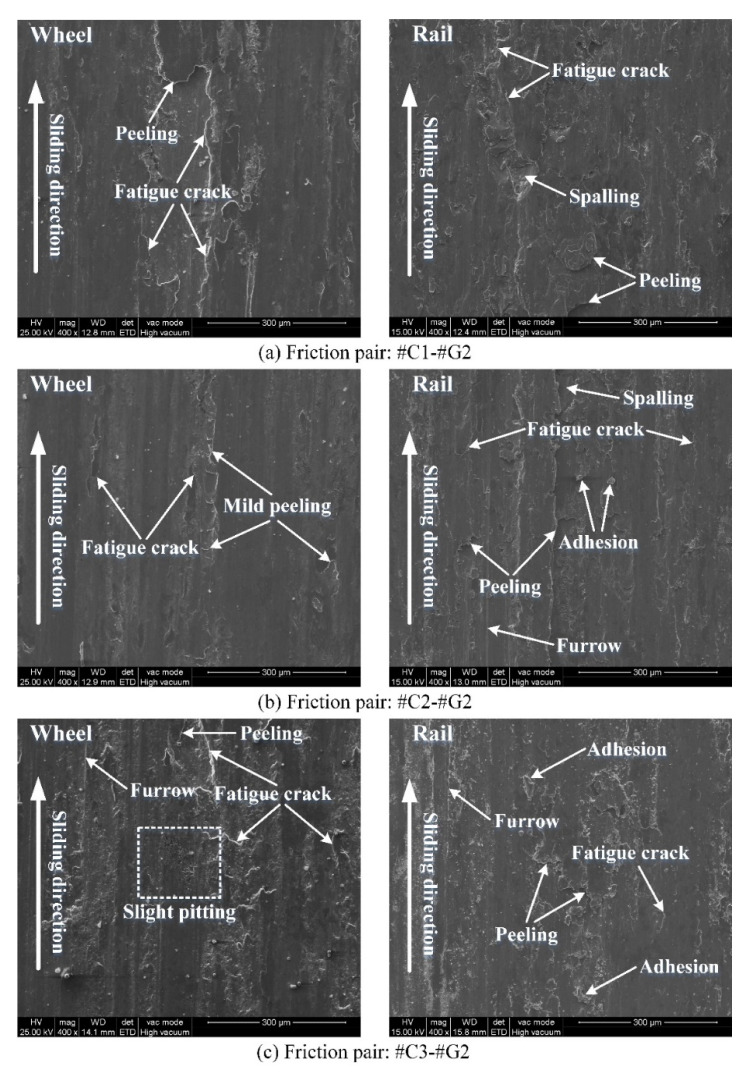
Morphology of worn surface of the wheel/rail samples under varied tribological pairs after the sliding contact: (**a**) #C1-#G2; (**b**) #C2-#G2; (**c**) #C3-#G2.

**Figure 13:**
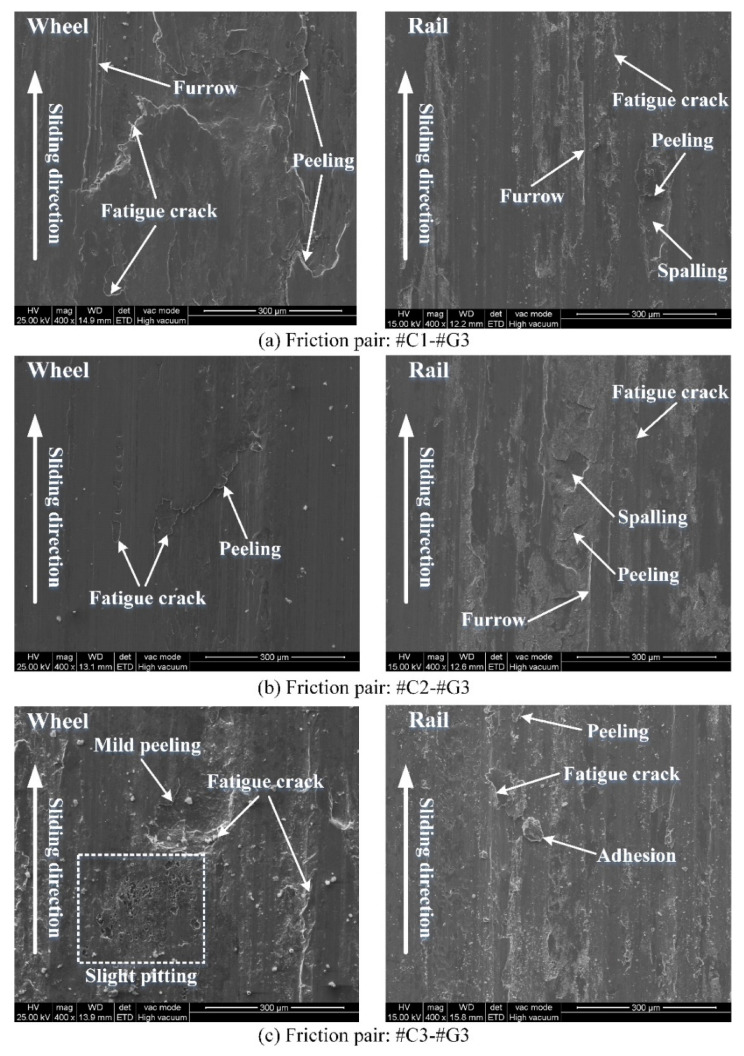
Morphology of worn surface of the wheel/rail samples under varied tribological pairs after the sliding contact: (**a**) #C1-#G3; (**b**) #C2-#G3; (**c**) #C3-#G3.

Figure 12 displays the worn surface morphology of the wheel/rail samples under tribological pairs #C1-#G2, #C2-#G2 and #C3-#G2. The worn surface morphology of the wheel/rail samples under tribological pairs #C1-#G3, #C2-#G3 and #C3-#G3 is shown in Figure 13. Likewise, there are discrepancies on the surface morphology between the wheel rings and rail blocks. The surface damage morphology presented in Figure 12 and Figure 13 is generally consistent with that displayed in Figure 11. Compared with Figure 11, Figure 12 and Figure 13, after pairing with the wheel rings turned with the same parameters, it can be observed that the worn surface of #G1 is rougher than that of #G2 and #G3, and the worn surface morphology of #G1 primarily presents widely and densely-distributed fatigue cracks, furrow and adhesion, while relatively sparsely-distributed damage can be observed on the surface of #G2 and #G3, which shows that the surface microhardness of the ground rail blocks has a direct bearing on the worn surface morphology. During the sliding process, the surface material of the rail block is continuously extruded and sheared under the influence of normal and tangential load, and the surface micro-protrusions of the wheel ring and the abrasive dusts trapped in the wheel/rail contact interface consecutively plough the rail block surface, finally generating furrows on the worn surface of the rail blocks easily. From the elucidation mentioned above, it can be demonstrated that the surface integrity of the machined wheel/rail samples influences the generation of the worn surface morphology after sliding.

### 3.4. Subsurface Plastic Deformation (SPD) Features

**Figure 14:**
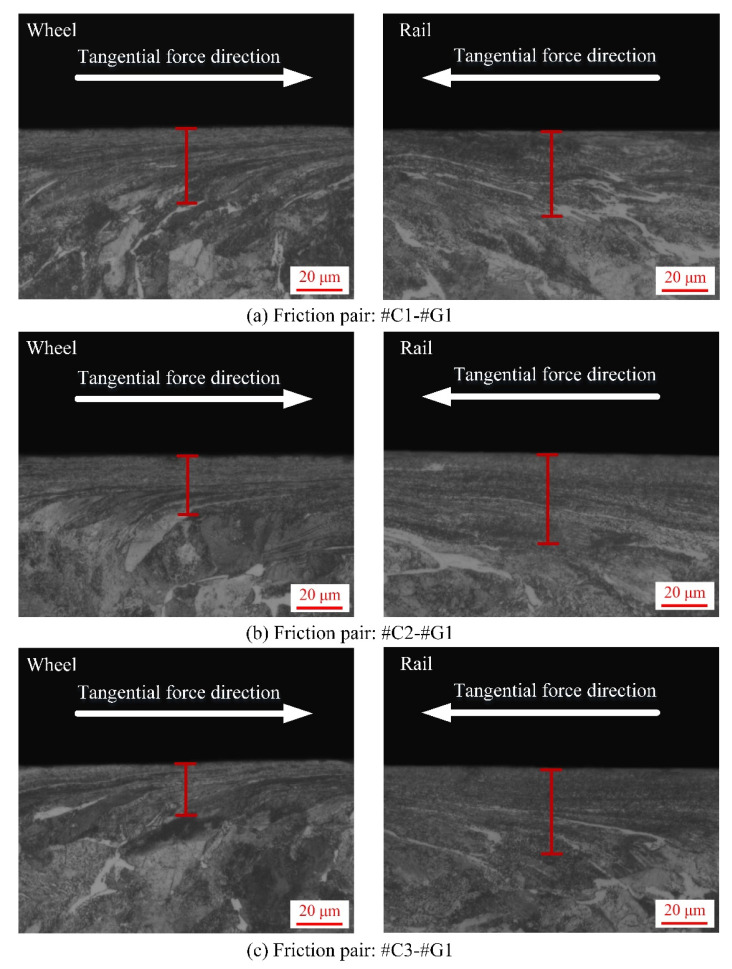
Micrographs of SPD of the machined wheel rings and rail blocks under various tribological pairs after the sliding contact: (**a**) #C1-#G1; (**b**) #C2-#G1; (**c**) #C3-#G1.

**Figure 15:**
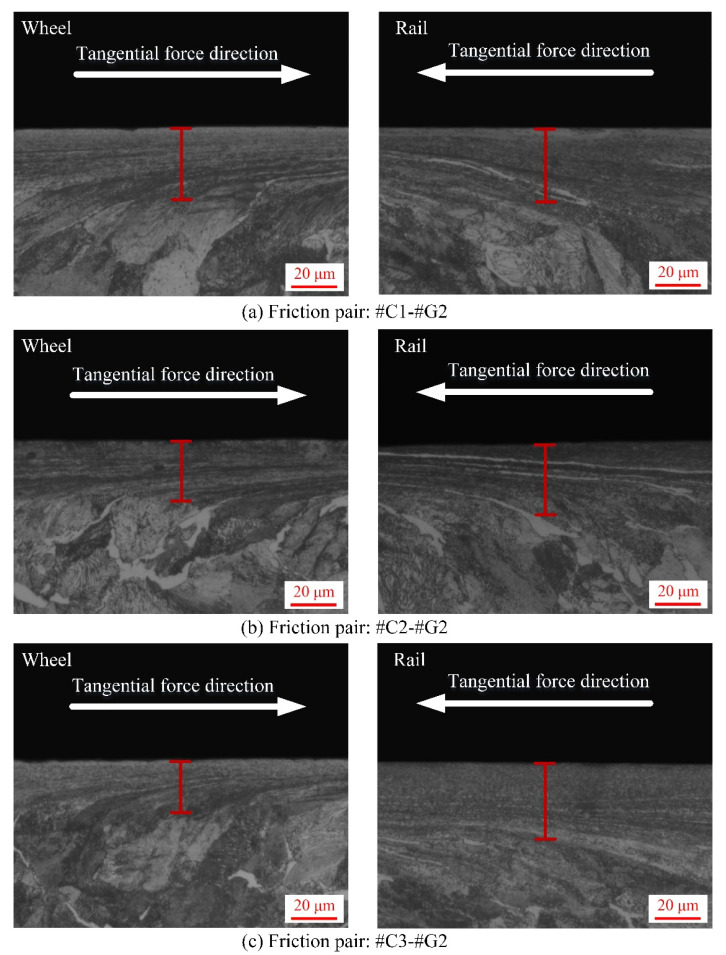
Micrographs of SPD of the machined wheel rings and rail blocks under various tribological pairs after the sliding contact: (**a**) #C1-#G2; (**b**) #C2-#G2; (**c**) #C3-#G2.

**Figure 16:**
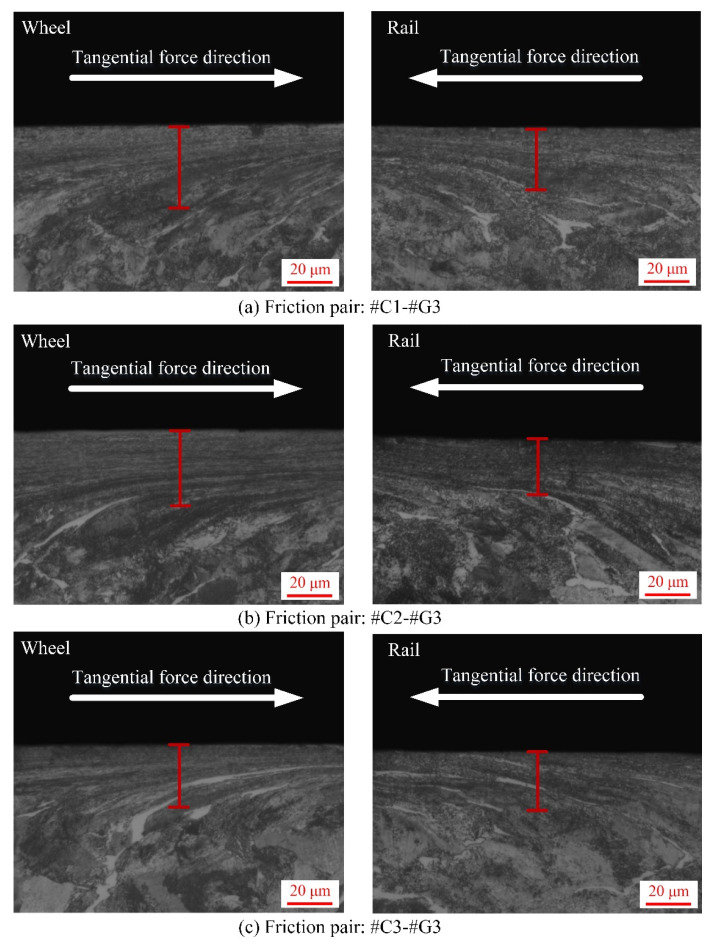
Micrographs of SPD of the machined wheel rings and rail blocks under various tribological pairs after the sliding contact: (**a**) #C1-#G3; (**b**) #C2#G3; (**c**) #C3-#G3.

After each sliding test, the sampling preparation for SPD characterization of wheel/rail specimens is illustrated in Figure 6. The SPD micrographs of the wheel rings and rail blocks after the sliding contact are displayed in Figure 14, Figure 15 and Figure 16, from which it can be found that various degrees of SPD of wheel/rail samples take place after sliding. The occurrence of SPD represents the presence of a work hardening phenomenon during the sliding process, ultimately enhancing the surface microhardness of the pairing samples. Within the deformation region, the wheel/rail metallographic microstructures are extended and refined along the sliding direction, and eventually the subsurface fibrous layer takes shape. According to the thickness of the SPDL of wheel/rail samples under dry rolling-sliding condition reported in our previous work [22], the thickness of the SPDL of the wheel samples after the rolling-sliding test ranges from 50 to 60 µm, while the thickness of the SPDL of the rail specimens is greater than 85 µm. In this current study, the thickness of the SPDL of the wheel rings falls in between 28 and 37 µm, while the thickness of the SPDL the rail blocks ranges from 21 to 42 µm. It can be found that whether it is under the dry sliding condition or under the dry rolling-sliding condition, the thickness of the SPDL of all the wheel/rail samples shows an increasing tendency after each friction and wear test. In general, compared with the thickness of the SPDL of the machined wheel/rail samples prior to the sliding test, the thickness increase degree of the SPDL of the rail blocks is greater than that of the wheel rings. This is caused by the rail block making continuous contact with the wheel ring, which makes the accumulation of plastic deformation easy to occur on the subsurface of rail blocks under the influence of the unidirectional frictional force. Some fixed position on the circumferential surface of the wheel ring merely makes periodic and intermittent contact with the rail block, and the subsurface plastic deformation is distributed throughout the whole circumferential surface of the wheel ring, which causes weak plastic deformation accumulation effect. After pairing with the rail blocks ground with the same parameters, the thickness of the SPDL of #C2 and #C3 is smaller than that of #C1, and the thickness of the SPDL of #C1 is the largest, which reveals that when the surface microhardness and surface roughness of the turned wheel rings are comparatively small, a thicker SPDL is generated after sliding. After pairing with #C1, #C2 and #C3, no apparent discrepancy on the thickness of the SPDL of #G1 can be found. From Figure 14, Figure 15 and Figure 16, it is also observed that there is no much difference on the thickness of the SPDL of #C2 and #C3, which means that when the values of surface roughness and microhardness of the turned wheel rings increase within the certain range simultaneously, the thickness of the SPDL does not alter remarkably after pairing with the rail blocks. Compared with Figure 14, Figure 15 and Figure 16, it can be observed that after pairing with the wheel rings turned with the same parameters, the thickness of the SPDL of #G1 is greater than that of #G2 and #G3, which indicates that when the surface microhardness of the ground rail blocks is comparatively small, a thicker SPDL is formed after pairing with the wheel rings.

From the aforementioned detailed analyses and the corresponding elucidation of the experimental results of the wear and damage behaviors of machined wheel/rail materials under dry sliding condition, it can be clearly seen that the machining parameters of the wheel/rail specimens prior to the sliding test exert a significant impact on the wear and damage characteristics of wheel/rail materials. From the perspective of the total wear loss of wheel/rail materials among all the tribological pairs and the damage degree of the wheel samples, the most preferable wear-resistance property is reached when #C2 makes the sliding contact with the rail discs, which means that the most appropriate machining parameters of the wheel samples in this study is the parameter combination of cutting speed *v*_c_ = 70 m/min, feed speed *f* = 0.6 mm/r and depth of cut *a*_p_ = 1.4 mm. This study can serve as a reference for the selection of wheel/rail machining parameters, which can ameliorate the wheel/rail interaction conditions and extend the wheel/rail service life span.

## 4. Conclusions

This work systematically investigated the wear and damage characteristics of the machined wheel/rail materials under dry sliding conditions by virtue of a block-on-ring tribometer. The following main findings can be summarized from this study:

1. Before the sliding test, the surface microhardness of the ground rail blocks is larger than that of wheel rings, while the thickness of the SPDL and surface roughness of the rail blocks are much smaller than those of wheel rings. After the sliding test, the surface microhardness of the wheel rings and rail blocks increases strikingly. The maximal surface microhardness increment of the wheel rings is 45.2%, while the maximal surface microhardness increment of the rail blocks is 50.8%. The thickness of the SPDL of wheel rings after sliding falls in between 28 and 37 µm, while the thickness of the SPDL of rail blocks falls in between 21 and 42 µm. The thickness increase degree of the SPDL of rail blocks is larger than that of the wheel rings.
2. The variations of friction coefficients are classified into break-in phase and steady-wear phase. The wheel rings and rail blocks machined with various parameters engender different friction coefficients after pairing with each other, and the wheel rings with larger initial surface roughness give rise to larger friction coefficients. At the stable wear phase, the friction coefficients among different tribological pairs range from 0.32 ± 0.06 to 0.45 ± 0.06.
3. After every sliding experiment, the wear loss of the rail material is more than 0.1581 g, while the wear loss of the wheel material falls in between 0.0163 and 0.0283 g. The wear loss of rail material is much larger, which means that the wheel material exhibits more excellent wear-resistance property. The surface microhardness, roughness and SPD of the machined wheel/rail samples impose a combined influence on the tribological behavior. The tribological pair with proper initial surface roughness and microhardness can engender the smallest amount of total wear loss. In terms of the total wear loss of wheel/rail materials among all the tribological pairs, the comprehensively preferable wear-resistance performance is achieved as #C2 pairs with the rail blocks.
4. The surface damage morphologies between the rail blocks and wheel rings after sliding are different. By and large, more serious surface damage of the rail blocks is observed. The worn surface morphology of the wheel rings primarily presents peeling, adhesive wear and fatigue cracks, while the worn surface morphology exhibits the combination of spalling, furrow wear, peeling, fatigue cracks and various degrees of adhesion.

## Figures and Tables

**Table 1:** Chemical components of wheel/rail materials (wt.%).

Materials	C	Mn	Si	S	P	Fe
Rail	0.65–0.76	0.70–1.20	0.15–0.58	≤0.025	≤0.030	Balance
Wheel	0.55–0.65	0.50–0.80	0.17–0.37	≤0.025	≤0.025	Balance

**Table 2:** Grinding parameters of the rail workpiece.

Experiment Number	Wheel Peripheral Speed *v*_s_ (m/s)	Workpiece Velocity *v*_w_ (m/min)	Grinding Depth *a*_e_ (mm)
#G1	30	0.3	0.05
#G2	30	0.4	0.07
#G3	30	0.5	0.09

**Table 3:** Turning parameters of the wheel rollers. [22].

Experiment Number	Cutting Speed *v*_c_ (m/min)	Feed Speed *f* (mm/r)	Depth of Cut *a*_p_ (mm)
#C1	70	0.4	1.2
#C2	70	0.6	1.4
#C3	70	0.8	1.6

**Table 4:** Surface roughness and hardness of the machined wheel/rail samples.

Experiment Number	Surface Roughness *R*_a_ (μm)	Surface Microhardness (HV_0.1_)
#C1	3.02	324.48
#C2	4.18	342.35
#C3	6.41	356.72
#G1	1.58	365.12
#G2	1.69	377.23
#G3	1.83	390.41

## Data Availability

The data presented in this study are available from the corresponding author upon request.
